# Potential Applications of Genome-Wide Association Studies in Establishing Climate Resilience in Livestock: A Comprehensive Review

**DOI:** 10.3390/ijms27083498

**Published:** 2026-04-14

**Authors:** Gajendirane Kalaignazhal, Mullakkalparambil Velayudhan Silpa, Chinmoy Mishra, Ebenezer Binuni Rebez, Santhi Priya Voggu, Pasuvalingam Visha, Guru D. V. Pandiyan, Artabandhu Sahoo, Christopher Browne, Umberto Bernabucci, Frank Rowland Dunshea, Veerasamy Sejian

**Affiliations:** 1 College of Veterinary Science and Animal Husbandry, Odisha University of Agriculture and Technology, Bhubaneshwar 751003, India; gnazhal99@gmail.com (G.K.); drchinmoymishra@gmail.com (C.M.); 2 Department of Animal Genetics and Breeding, Rajiv Gandhi Institute of Veterinary Education and Research, Kurumbapet, Puducherry 605009, India; mv.silpa@gmail.com; 3 Department of Agricultural and Forestry Sciences, The University of Tuscia, 01100 Viterbo, Italy; binunirebez.e@gmail.com (E.B.R.); bernab@unitus.it (U.B.); 4 Department of Animal Science, University of Connecticut, Storrs, CT 06269, USA; 5 Department of Veterinary Physiology and Biochemistry, Veterinary College and Research Institute, Tamil Nadu Veterinary and Animal Sciences University, Salem 636112, India; drpvisha@gmail.com; 6 Department of Veterinary Physiology, Madras Veterinary College, Tamil Nadu Veterinary and Animal Sciences University, Chennai 600007, India; gurupandy@gmail.com; 7 Centre for Climate Resilient Animal Adaptation Studies, ICAR-National Institute of Animal Nutrition and Physiology, Adugodi, Bangalore 560030, India; sahooarta1@gmail.com; 8 Compassion in World Farming International, Surrey GU71EZ, UK; christopher.browne@ciwf.org; 9 Faculty of Science, School of Agriculture, Food and Ecosystem Sciences, The University of Melbourne, Parkville, Melbourne, VIC 3010, Australia; fdunshea@unimelb.edu.au; 10 Faculty of Biological Sciences, School of Biology, The University of Leeds, Leeds LS2 9JT, UK

**Keywords:** adaptation, biomarkers, climate resilience, GWAS, livestock breeding, sustainability

## Abstract

Given livestock’s crucial role in global food security and economic stability, the alarming threat of climate change calls for the implementation of effective mitigation strategies for climate-resilient livestock production. Management and nutritional strategies offer temporary relief, whereas genetic approaches represent a permanent solution. The role of genetic tools in enabling the development of climate-resilient livestock breeds is widely recognized. Genetic tools like microarrays, RNA-seq, omics, and GWAS can improve the understanding of livestock’s climate adaptability at a molecular level. These tools facilitate the identification of biomarkers for thermo-tolerance, bordering on climate-resilient livestock breeding. Among them, studies employing genome-wide association studies (GWAS) have increased in recent years. GWAS have the potential to improve the genetic basis of thermo-tolerance in heat-stressed livestock populations. GWAS have been used to identify candidate genes for complex and economically important traits in livestock. These include growth, reproduction, disease resistance, milk, meat, and wool production traits under heat stress conditions. This makes GWAS a useful tool for identifying biomarkers that can be incorporated in breeding programs through marker-assisted selection (MAS). The integration of these potential biomarkers into selection and breeding programs would allow GWAS to substantially refine breeding strategies, thereby advancing the climate-resilient potential and sustainability of the livestock sector. Furthermore, GWAS, when utilized along with emerging technologies like Artificial Intelligence (AI), machine learning (ML), and deep learning (DL) for genomic prediction, can predict genetic aspects of livestock adaptation more efficiently and precisely. Thus, future studies should focus on integrated modeling approaches for improving the climate resilience of livestock without jeopardizing their production potential. Such an effort will contribute to sustainable livestock production as well as ensure food security for the growing human population amid changing climate conditions.

## 1. Introduction

Climate change has become a significant concern in the livestock sector globally, impacting the productivity and reproductive health of animals. Among the various climatic stressors, heat stress is the biggest threat, having a detrimental impact on the production, reproduction, and welfare of animals through their direct and indirect effects [1]. The livestock sector, apart from being severely impacted by the effects of climate change, is also a contributor to greenhouse gases, leading to global climate change. It is estimated that the livestock sector alone accounts for about 14.5% of the total anthropogenic greenhouse gas emissions [2]. Therefore, it is imperative to develop climate-resilient animals that can withstand extreme climatic conditions while having low methane emission traits without compromising their productive and reproductive potential. This will help to ensure sustainable livestock production to feed the growing human population, which is projected to grow to 9.7 billion and 10.9 billion by 2050 and 2100, respectively [3].

Genetic tools provide a long-term solution for identifying biomarkers of thermo-tolerance that will help in developing breeds that are resistant to climate change. The striking advancement and development of next-generation molecular tools have prompted research into genome-wide association studies (GWAS). GWAS use single-nucleotide polymorphisms (SNPs) as genetic markers to investigate the association between genetic variations and traits across an entire genome. This linkage disequilibrium (LD)-based approach identifies candidate genes and quantitative trait loci (QTL) influencing phenotypes and gives insight into their molecular mechanisms [4].

**Figure 1:**
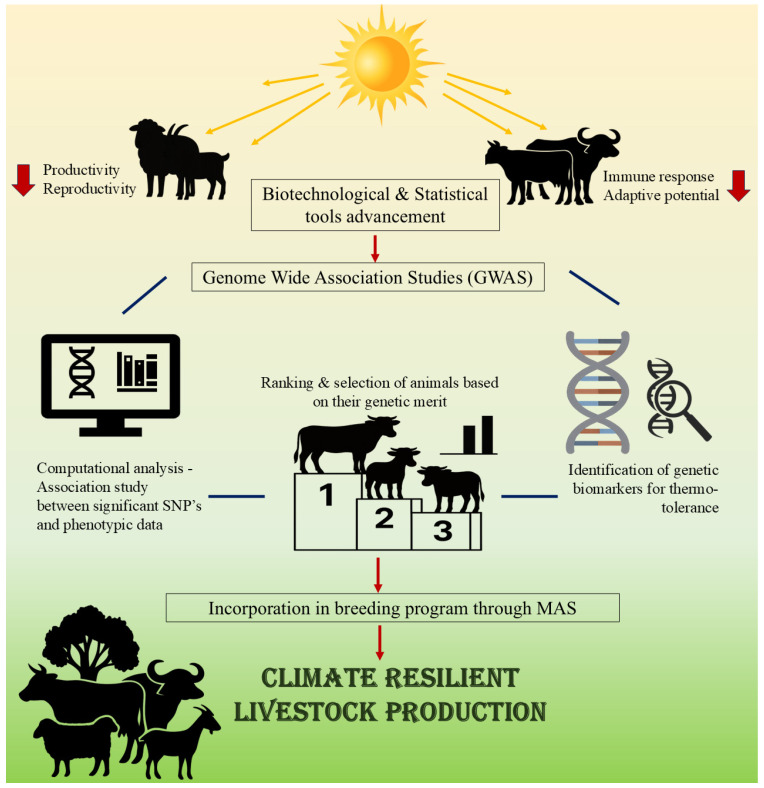
Role of GWAS for climate-resilient livestock production.

Several GWAS have been carried out and have identified candidate genes for low methane emission traits [5], thermo-tolerance linked to milk production traits [6], reproduction traits [7], immune response traits [8], and adaptive traits [9] in livestock to make them climate-resilient. The candidate genes can subsequently be employed as biomarkers to enable the selection of better animals through genetic selection in future breeding strategies. Subsequent research can help explore the use of these technologies to redefine breeding strategy through marker-assisted selection and genomic selection [10]. However, there are certain limitations and challenges associated with GWAS in livestock that could result in erroneous associations and elevated errors during association tests, which need to be addressed to generate meaningful and valuable results [11]. Figure 1 illustrates the role of GWAS for climate-resilient livestock production.

Additionally, with advancements in genetic technologies, large datasets, or “big data,” are generated daily, requiring efficient management. Artificial Intelligence (AI) and machine learning (ML) systems can analyze these large datasets [12], helping predict genetic traits related to adaptation to different environmental conditions. AI and ML are being utilized to derive valuable insights from datasets, surpassing the constraints of traditional linear models [13]. Their adaptability and capacity to identify patterns in large, noisy datasets make them essential for processing and analyzing complex data derived from these statistical tools, thereby enhancing efficiency and productivity while also reducing the likelihood of human error [14]. Therefore, this literature review provides a synthesis of the recent progress in assessing the climate resilience of livestock using various genomic models and possibly the application of GWAS in developing climate resilience in livestock. Also, it delves into the benefits of AI and ML in evaluating heat stress in livestock, highlighting their potential to complement the increasing global food needs through enhanced resilience and production of livestock despite climate change.

## 2. Climate Change and Livestock Production: Importance of Identifying Climate-Resilient Livestock Breeds

Climate change has emerged as a major threat due to the rise in global temperatures, which must be kept to 1.5 °C to avoid biodiversity collapse [15] and extreme weather (REF), food insecurity (REF), and mass extinction [16].

The livestock sector, especially animals in the tropical and sub-tropical countries, is experiencing lower productive and reproductive efficiency. This is due to the multiple environmental stressors experienced by the animals through the direct and indirect effects of climate change [17,18,19]. Among the various climatic stressors, heat stress is the most dangerous, as it induces the body to undergo drastic biological changes, resulting in adverse impacts on the productivity, reproductivity, and welfare of the animals [20]. Heat stress results in a slower growth rate, a reduction in the quality and quantity of milk and meat, decreased fertility, increased veterinary expenses, threatens food security, and results in huge economic loss [21,22]. However, the global population continues to grow rapidly, resulting in increased overall food demand. Especially, the livestock sector plays a significant role in satisfying global food demands, and the demand for livestock products is expected to increase by 70% by 2050 [23]. Therefore, suitable measures must be taken to increase livestock productivity amidst the changing climatic conditions to promote sustainable livestock production and support food security.

This is possible by identifying climate-resilient breeds with inherent resilience and adaptive capacity that can cope with the harsh climatic conditions without jeopardizing their productive and reproductive potential [24,25]. However, the resilience potential of the livestock differs across species, breed, and individuals because of the genetic variation among them [26]. Indigenous livestock breeds in the tropical and sub-tropical regions have better thermo-tolerance potential than the temperate breeds. These indigenous livestock breeds, despite having less productive potential than the exotic breeds, easily adapt to adverse weather conditions due to their enhanced adaptive potential [27,28,29]. This emphasizes the importance of identifying climate-resilient livestock breeds that will, in turn, help with the economic growth of the country as well as satisfy the food demands of the growing human population, thereby ensuring food security.

## 3. Progression Towards GWAS for Economically Important Traits in Livestock

Economic traits are those that have an impact on the income obtained or the cost of production. Economic traits in livestock directly influence the profitability and efficiency of livestock production [30]. Some of the economically important traits are growth traits, milk, meat, and wool production traits, reproduction traits, and disease resistance traits. Selection for these traits in livestock will improve the production potential of the animals, which will boost the economic returns to the farmers [31]. The degree to which economic traits can be enhanced by selective breeding depends on the heritability of these traits, which is different for each trait. In general, traits that have moderate to high heritability can be reworked through genetic improvement. However, maximizing production and profitability for low heritable traits requires a multifaceted approach that integrates genetic, management, and environmental strategies. Therefore, over time, researchers and breeders have increasingly explored shifts in livestock breeding programs aimed at producing more robust animals.

Traditional methods of livestock breeding have focused on phenotypic visual evaluations and basic performance indicators, allowing breeders to select superior animals for breeding. However, traditional methods are not efficient at identifying traits with low heritability, which are difficult and expensive to measure [32]. These issues have been overcome with the development of genomic selection. Genomic selection utilizes DNA markers to predict the genetic potential of animals, enabling more accurate and accelerated improvement of economically important traits [33]. By analyzing the genome rather than just observable characteristics, breeders can identify and select animals with superior genetic potential for traits such as growth rate, feed efficiency, and disease resistance. This approach significantly enhances the accuracy of selection and reduces the time required to make genetic improvements by facilitating the selection of animals at an early age, enabling breeding strategies to maximize genetic advancement while reducing the expenses [34].

**Figure 2:**
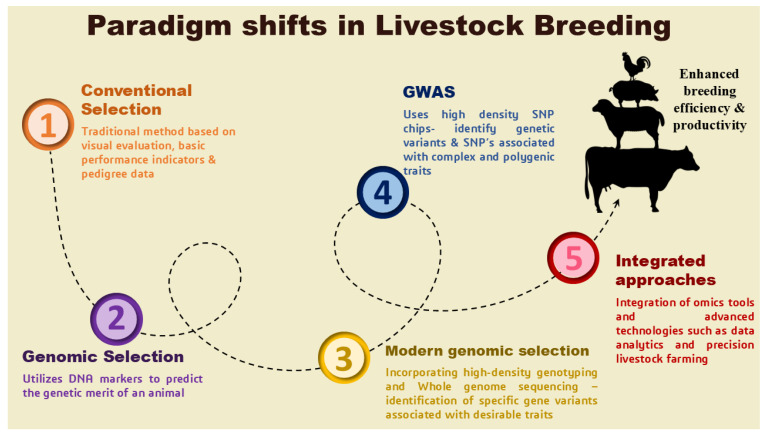
Paradigm shifts in livestock breeding programs for producing robust animals.

Modern genomic selection incorporates high-density genotyping and whole-genome sequencing, providing detailed genetic information that enables the identification of specific gene variants associated with desirable traits [32]. This enables breeders to make better decisions during selection and implement targeted breeding strategies. Additionally, genomic selection supports multi-trait improvement by simultaneously addressing several economic traits, such as milk yield, carcass quality, and reproductive performance [35]. The availability of genome sequences and identification of SNPs for domestic animals led to the expansion of GWAS to the livestock sector [36]. GWAS are a powerful tool for identifying genetic variants linked to complex and economically important traits in livestock. Several GWAS have been conducted in livestock to identify candidate genes associated with economically important traits, including growth traits [37], milk production [38], meat production [39], wool production [40], reproduction [41], and disease resistance traits [42]. The identified candidate genes can be used as biomarkers in marker-assisted selection, thereby refining the breeding strategies to improve animal performance. Figure 2 describes the paradigm shifts in livestock breeding programs for producing robust animals. In addition, the recent integration of genomic tools with other advanced technologies, such as data analytics and precision livestock farming, has been shown to further enhance breeding efficiency and productivity [43]. Overall, genomic selection has emerged as a key component of modern livestock breeding programs, driving significant gains in performance while addressing both economic and environmental challenges.

## 4. Different Genetic Models for Establishing Climate Resilience in Livestock

**Figure 3:**
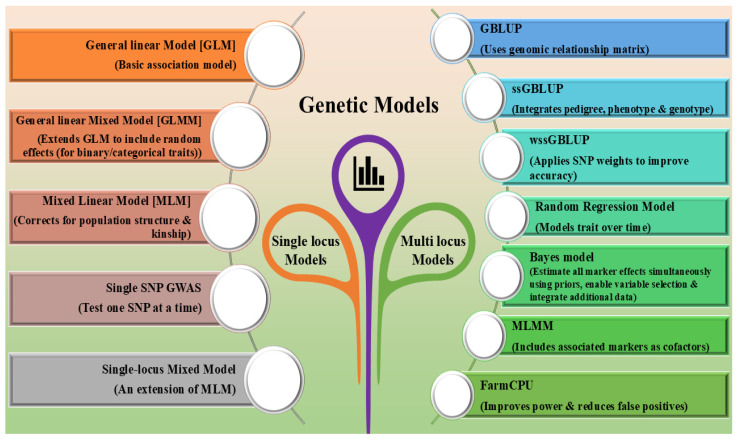
Different genetic models for establishing climate-resilient livestock production systems. GLM, General Linear Model; GLMM, General Linear Mixed Model; MLM, Mixed Linear Model; GBLUP, Genomic Best Linear Unbiased Prediction; ssGBLUP, Single-Step Genomic Best Linear Unbiased Prediction; wssGBLUP, Weighted Single-Step Genomic Best Linear Unbiased Prediction; MLMM, multi-locus mixed model; FarmCPU, Fixed and Random Model Circulating Probability Unification.

Heat stress was considered a major challenge in tropical livestock production systems, can be temporarily alleviated by managing housing and nutrition [44]. On the other hand, genetic selection for tolerance to heat can provide a cost-effective and sustainable measure, providing a long-term solution [45]. Traditional breeding methods, although helpful in the development of livestock, were time-consuming and did not always encompass the entire genetic diversity. However, with the advent of molecular technology and DNA markers, more precise and effective breeding techniques have been developed in farm animals [46]. To ensure that this approach works, it is essential to develop heat stress models that incorporate climate data into account to precisely assess and predict heat tolerance [47]. The genetic models discussed in this section can be grouped as (i) genomic selection models, which are employed for the prediction and selection of animals, and (ii) GWAS-based statistical models, which are employed for the identification of SNPs, candidate genes, and genomic regions for adaptive traits. Though the two approaches employ genomic information, the objectives of the two approaches differ. Figure 3 describes the various genetic models for establishing climate-resilient livestock production systems.

Genomic selection provides a prospect for the selection of animals based on their genomic Estimated Breeding Value (GEBV). Genomic Best Linear Unbiased Prediction (GBLUP) is widely applied in genomic selection to predict an animal’s breeding value based on a genomic relationship matrix [48]. For example, random regression models were used by Nguyen et al. [49] and Osei-Amponsah et al. [50] to measure heat tolerance as the rate of decline in production of animals due to heat stress, given a THI (temperature humidity index) value. Both studies indicated genomic selection based on GEBV for heat tolerance would enhance dairy cattle efficiency and resistance under heat stress.

Another commonly used model is the Single-Step Genomic Best Linear Unbiased Prediction (ssGBLUP), which is an enhanced version of GBLUP. By integrating pedigree, phenotypic, and genomic data in one step, ssGBLUP enhances the accuracy and efficiency of genetic evaluations while reducing bias in breeding value estimates [51]. Sungkhapreecha et al. [52] compared ssGBLUP and BLUP for genomic evaluation of milk yield and thermo-tolerance of Thai–Holstein cows. They concluded that ssGBLUP was more precise (0.36 vs. 0.18 for BLUP) and less inconsistent in its prediction [52]. A similar 25% improvement in prediction accuracy using ssGBLUP over BLUP was reported by Fragomeni et al. [53] in pigs. An extent of this ssGBLUP is the weighted ssGBLUP (WssGBLUP) that uses a weighted genomic relationship matrix (GRM) to account for both genomic data and pedigree-based relationships to estimate genetic merit more accurately. Luo et al. [54] performed a WssGWAS to detect QTL linked to heat stress markers (rectal temperature, respiration rate, and drooling score) of Holstein cattle. The model estimated SNP effects and weights, which validated the polygenic inheritance of heat tolerance [54]. Another study used a weighted single-step GWAS to estimate the SNP effects from genomic breeding values of single-step genomic BLUP (ssGBLUP) [55]. It found that this weighted ssGBLUP model slightly outperformed the base ssGBLUP and improved the accuracy of genetic predictions [55].

Linear models such as Generalized Linear Mixed Model (GLMMs) were also employed in these studies. The GLMM is an extension of the GLM by incorporating both fixed and random effects, making it useful for analyzing correlated, non-normal data, especially in clustered or longitudinal studies [56]. Recce et al. [57] followed the GLMM to analyze the impact of heat stress during gestation on the reproductive performance of Holstein cows. The results showed that exposure to high THI during the first trimester negatively affected reproductive efficiency, suggesting potential long-term epigenetic effects on offspring [57]. Random Regression Models (RRMs) are sophisticated statistical methods where genetic and environmental effects are modeled as random through time, enabling the estimation of breeding values with increased accuracy and a clear picture of the variation of biological traits and physiological processes across time [58,59].A study adopted an RRM with reaction norm functions to assess heat tolerance in multi-breed dairy cattle by analyzing milk production responses to different heat loads (THI 50 and THI 80) [60]. It identified resilience indicators, such as the slope and absolute value of the reaction norm, with cows having ≤50% *Bos taurus* genes and those in semi-arid environments showing the highest heat tolerance [60].

Several statistical models, including the General Linear Model (GLM), Mixed Linear Model (MLM), Bayesian model, single SNP model, single locus mixed model, random regression models, Bayesian models, PCA-based GWAS, Single-Step GWAS, Weighted Single-Step GWAS, and FarmCPU, are employed in GWAS to determine the climate resilience in livestock. Notably, Linear Mixed Models (LMMs), especially Mixed Linear Models (MLMs), are the preferred approach for GWAS due to their ability to control population structure and relatedness by the inclusion of fixed and random effects in the models. They have the effect of limiting false positive associations and enhancing the reliability of SNP identification. Compressed MLMs (CMLM), enriched CMLMs (ECMLM), and multi-locus mixed models (MLMMs) are extensions of the LMM approach. These have been proposed to improve power and efficiency by the inclusion of more than one locus or by the optimization of the estimation of variance component effects. Although these approaches are undoubtedly effective and robust, the limitations of these approaches in detecting small effect associations and the computationally intensive nature of these approaches are a limitation for their application to genomic-scale data analysis.

Models utilized in GWAS are primarily designed to detect associations between genetic variants (SNPs) and complex traits, such as climate resilience. These models are essential for identifying candidate genes, QTLs, and biological pathways underlying thermo-tolerance and adaptation. Cebeci et al. (2023) [61] identified different statistical models (Blink, FarmCPU, SUPER, MLMM, GLM, MLM, CMLM, and ECLMM) in GWAS based on the genotypic data of domesticated goats. Blink and FarmCPU were found to efficiently manage false positives for medium to high heritability traits. The multi-locus models, SUPER and MLMM, were superior to the single-locus methodologies (GLM, MLM, CMLM, and ECLMM), though the latter performed better than the old single-locus methods [61]. Zhang et al. [62] conducted a comparative analysis between single-trait GWAS and PCA-based GWAS to detect SNP associations in a different study. Principal component analysis (PCA) is another method that effectively increases multiple-trait GWAS by increasing statistical power for QTL detection. The research established that the PCA-based method identified pleiotropic quantitative trait nucleotides (QTNs) more meaningfully than the single-trait model, proving PCA-based multiple-trait GWAS increases statistical power for detecting QTL and exploring pleiotropic QTNs [62]. The conventional method for GWAS analysis often employs a Mixed Linear Model (MLM), in which polygenic effects are treated as random effects [63]. The MLM models are effective in controlling population structure and relatedness in GWAS [64]. A GWAS used a Mixed Linear Model (MLM) to identify SNPs related to resilience to weather stress, milk yield, and productive life in Chios dairy sheep [65]. Key genomic regions, particularly on chromosome 5, were associated with heat stress resilience, with genes linked to heat dissipation and immune responses [65]. To investigate heat stress in a Gir × Holstein F2 population, Otto et al. [66] employed a single-SNP GWAS methodology, which determines the association between a single genetic variant and the trait of interest. Six SNPs were highly associated with rectal temperature changes (ΔRT), and candidate genes like *LIF*, *OSM*, *TXNRD2*, and *DGCR8* were discovered. Alternatively, a single-locus mixed model is akin to a single-SNP model but adjusts for both fixed and random effects. A single-locus mixed model GWAS was employed in a study that identified a significant *OAR4* marker linked to the local adaptation of Greek sheep and found six candidate genes and two QTLs for fat tail deposition [67].

Bayesian procedures are multi-locus models that allow for the simultaneous estimation of the effects of all genotyped markers, with various prior distributions, variable selection to detect significant markers, and the inclusion of additional data [68]. Using a GWAS approach, Luna-Nevarez et al. [69] used single-marker analysis and Bayesian multi-marker models (Bayes C and Bayes B) to find genetic markers for heat stress tolerance in pregnant ewes. The Bayesian models revealed 26 chromosomal regions that account for 9.8% of the variation in heat stress tolerance [69]. Furthermore, a sophisticated model in GWAS is FarmCPU. It integrates Mixed Linear Models (MLMs) and stepwise regression, employing GLM for scanning markers and selecting pseudo-QTNs, followed by iterative fine-tuning based on the log-likelihood of a random effects model to enhance genetic analysis [70]. To improve fertility, a study was done on the genetic factors influencing the heat index and activity peak in Holstein cows [71]. Using FarmCPU software, the GWAS identified 31 related genes, including *PTGS1*, *NDUFA8*, and *VEGFA*, and seven significant SNPs related to cattle reproduction. Activity peak and heat index were found to have moderate heritability, suggesting that they may improve dairy cow fertility [71]. Therefore, there are several genetic models that are employed, and the models are selected based on the particularities of the dataset, the available computing capacity, and the complexity of the dataset. Each model has its strengths and weaknesses, and the model selection significantly affects the accuracy and performance of genetic improvement [72]. Thus, model selection is important, as models used in genomic selection enhance the prediction and selection of resilient animals, while GWAS models facilitate the discovery of genetic variants underlying climate adaptability. Clearly distinguishing these roles improves the understanding and effective application of genetic tools in developing climate-resilient livestock. Table 1 provides a summary of genetic models for establishing climate resilience in livestock.

## 5. Applications of Genomic Models in Establishing Phenotypic Plasticity in Livestock

The ability of a genotype to produce distinct phenotypes in response to changing environmental, biotic, or abiotic conditions is known as phenotypic plasticity [73]. By influencing and changing their genetic expression in response to environmental factors, it plays a critical role in helping animals adapt to climate change [74]. Phenotypic plasticity is illustrated by reaction norms, where different genotypes may respond differently to environmental changes, with the genetic variation in these responses determining evolutionary outcomes [75]. By integrating genotypic data with reaction norm models, the accuracy of evaluating genotype-by-environment (GxE) interactions is significantly improved compared to traditional pedigree-based analysis. Table 2 describes the various GWAS models used for identifying biomarkers governing various functions using different whole-genome sequencing.

Genetic selection for phenotypic plasticity offers an effective strategy for developing animals with enhanced adaptability to diverse climatic conditions [76]. In a study by Mateescu et al. [77], where they assessed body temperature plasticity under heat stress across six crossbred groups, they emphasized the need for effective strategies to identify genes associated with thermo-tolerance to improve their resilience. To achieve this, innovative molecular and statistical approaches, including microarrays, RNA-seq, omics, and GWAS, have enabled the investigation of plasticity at the molecular level. Through this, it shows how environmental factors interact with genetic variation to play a role in phenotypic plasticity and adaptive characters [78]. Once reaction norms are integrated with sophisticated genomic models, it helps to detect genomic regions linked to animal adaptation under different environmental conditions [79].

Environmental change regulates gene expression across various molecular and functional categories. Several studies have shown that the combined high-throughput gene expression analysis over different environmental conditions with forward genetic strategies (i.e., eQTL mapping and GWAS of gene expression) can identify the genetic underpinnings of environmentally regulated gene expression. These studies have established correlations between genotype variation and environmental modulation of gene expression, enabling the identification of GxE interactions and distinguishing the genomic characteristics of genes with these interactions from those without them [80]. Furthermore, findings from such advanced technologies can be further analyzed through pathway and gene networks that can identify the underlying mechanisms behind the plasticity. Future studies investigating gene expression across multiple species and environmental cues may reveal shared patterns and effector genes in developmental plasticity [80].

Finally, the determination of the molecular processes driving the plastic responses of an organism to environmental changes will be important for determining the role that plasticity plays in evolution and how it contributes to evolutionary processes [81]. Such research enables scientists to find genes and biomarkers associated with traits that have plasticity and that can be incorporated into animal breeding schemes [82]. Plasticity, therefore, can develop through direct selection and as a correlated response to the selection for certain characteristics, and what is required is to include this possibility in breeding objectives for livestock, particularly ruminants [74].

## 6. Applications of Artificial Intelligence and Machine Learning in Genomics Approaches to Establish Climate Resilience in Livestock

Advancements in genomic approaches have enhanced our understanding of the complex mechanisms behind thermo-tolerance. However, with advancements in genetic technologies, large datasets, or “big data,” are generated daily, requiring efficient management. Artificial Intelligence (AI) and machine learning (ML) can process these large datasets [12], which assist in predicting genetic aspects of adaptation towards various environmental conditions [13]. Their adaptability and capacity to identify patterns in large, noisy datasets make them essential for processing and analyzing complex data [14]. Thus, the applications of AI and ML in genomic and genetic approaches, such as genomic selection, whole-genome sequencing, RNA sequencing, QTL mapping, GWAS, selection signatures, etc., will offer a promising path forward for developing effective breeding programs.

Genomic selection, an innovative way of assessing genetic performance, is gaining popularity because of its benefits over conventional techniques. Numerous studies conducted over the last few years have demonstrated the effectiveness of genetic selection in enhancing the robustness, welfare, and productivity of heat-stressed animals [83,84]. In genomics, these AI technologies can hasten genomic selection by improving DNA analysis through increased speed, accuracy, and cost-effectiveness and can also resolve challenges faced in genomic studies [85]. Machine learning is increasingly utilized in livestock genomic prediction due to its ability to uncover patterns in large, unstructured datasets, allowing for more accurate predictions of breeding values by combining genomic, phenotypic, and biological data [86].

Next-generation sequencing technologies, such as whole-genome sequencing, enable detailed analysis of genetic variations, such as SNPs, INDELs, CNVs, and SVs, while RNA sequencing (RNA-seq) offers improved resolution and reproducibility to analyze gene expression [87]. Machine learning and deep learning (DL) methods are promising tools for analyzing next-generation sequencing (NGS) data. However, care should be taken not to overfit since DL models can suffer from poor generalization across sequencing technologies or species [88].

GWAS detect SNPs correlated with a specific phenotype within a particular population. Numerous studies have reported applications of GWAS in mapping quantitative trait loci and detecting candidate genes and genomic areas associated with climate resilience. Through machine learning algorithms, it is possible to generate precise prediction models in high-dimensional data, which will improve our knowledge of genetic variations and their effects when used in conjunction with conventional GWAS approaches [89]. Such sophisticated ML methods, coupled with genomic information, can enhance genetic performance assessment under heat stress and aid in developing breeding programs for thermo-tolerance. Another important statistical application extensively used in livestock studies to maximize climate resilience is the identification of selection signatures, which assists in determining loci and candidate genes subjected to positive selection and contribute to adaptation mechanisms. Population genetic models using traditional statistical approaches can give misleading outcomes when assumptions have been broken, but ML presents an encouraging alternative by formulating the identification of signatures of selection as a classification problem [90,91]. The combination of ML with population genomics data promises much for breeding livestock that will be more resilient to climate adversity.

Advancements in DL and GPU technology have facilitated the integration of environmental variables with big multi-omics data, enhancing the potential of AI in genetic evaluations [92]. While AI is increasingly used in genomic approaches with various ML algorithms, there is a paucity of information on its application in evaluating heat-stressed ruminants [13]. However, AI’s role in this area is expected to grow as these technologies can help improve performance in harsh environments by selecting traits that enhance resistance to heat stress. To implement AI’s full capability, more research must be conducted to determine AI-based genomic methods and genetic assessment procedures for heat-stressed animals to improve their resilience potential. Overall, AI is a strong utility for providing greater efficiency and productivity, and there are fewer chances of human mistakes. Table 3 provides a comparison of different genomic models and AI/ML approaches for predicting phenotypes and climate adaptability in livestock.

## 7. Applications for GWAS in Mapping QTL of Different Important Functional Traits

The GWAS is a valuable tool for identifying genetic variations associated with important functional traits in livestock. These studies enable QTL mapping, or genomic regions associated with complex traits influenced by many genes, more easily [11]. GWAS are widely used for the identification of single-nucleotide polymorphisms (SNPs) associated with traits of interest; however, there is a general misunderstanding of LD block analysis and haplotype analysis. Linkage disequilibrium (LD) block analysis is a tool that identifies genomic areas where SNPs are inherited together. It provides a framework for understanding the genomic context of GWAS results and fine-tunes the genomic areas of interest. Haplotype analysis tests alleles at multiple associated loci within the genomic areas of interest. It provides a tool for understanding the joint effects of loci and the identification of complex trait associations. GWAS, LD block analysis, and haplotype analysis are tools that must be used sequentially for effective identification of genetic variants associated with complex traits.

**Figure 4:**
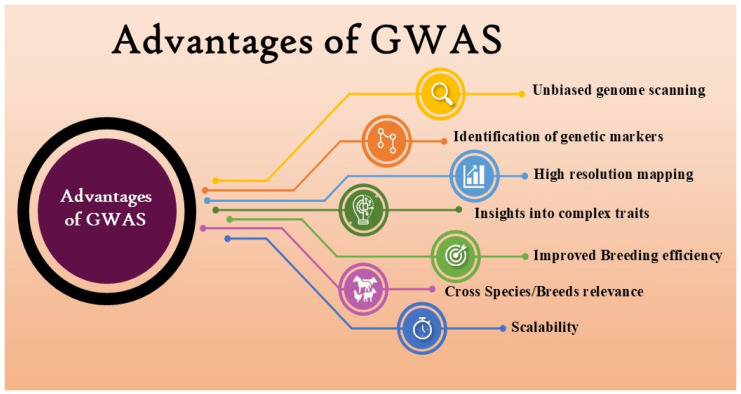
The various advantages associated with GWAS in the livestock sector.

However, the present review focuses specifically on GWAS, providing useful information on the molecular mechanisms of complex traits, making more effective and targeted breeding strategies possible to improve livestock productivity, health, and welfare. The GWAS performs association tests and identifies genes or regulatory factors that are important to the traits of interest, utilizing genomic sequence variations, predominantly single-nucleotide polymorphisms, along with phenotypic and pedigree data [93]. When compared to traditional QTL mapping methods, GWAS have several advantages. They can detect more targeted genomic regions harboring causative variations and have the capacity to detect causal variants with subtle effects [93]. Successful implementation is dependent on accurate phenotyping, coordination with genomic selection, and additional confirmation of recognized QTL for the purpose of practical usage in enhancing livestock productivity and well-being [94]. The findings will certainly contribute to improved breeding programs and comprehension of the genetic structure of complicated traits in domesticated animals. Several GWAS have been carried out in livestock for the mapping of QTL of various important characteristics, such as production traits, reproduction traits, immune response traits, adaptation traits, and low methane emission traits. Figure 4 highlights the various advantages associated with GWAS in the livestock sector.

### 7.1. Production Traits

Heat stress in milking animals occurs when the THI exceeds 68, resulting in lower milk yield and milk constituents [95]. The exotic and crossbred dairy cattle are most susceptible to heat stress. Reports have shown a decrease in milk production by 10 to 40% under repeated heat exposure due to higher metabolic turnover and increased milk production [1,95,96]. The effect of heat stress on milk production varies with factors including lactation, heat intensity, and the genetic milk production ability of the cow [96]. The GWAS has become a key method in the identification of QTLs and chromosomal loci associated with traits of milk production, such as milk yield [6,97,98,99], fatty acids in milk [100], fat, and protein percentage [97,99].

Zamorano-Algandar et al. [6] and Velayudhan et al. [98] performed GWAS to identify SNPs linked with milk production traits in dairy cows. Zamorano-Algandar et al. found six SNPs for total milk yield within the genes *TLR4*, *GRM8*, and *SMAD3*. The identified genes were linked to insulin signaling and metabolic regulation, which indicated that variation in energy utilization pathways contributes to divergent production performance in heat-challenged animals [6]. Likewise, Velayudhan et al. [98] discovered two SNPs (rs109340659 and rs41571523) to be significantly associated with test-day milk yield, along with possible candidate genes *FBRSL* and *CACN* in proximity to these SNPs, which have been implicated in milk yield. Similarly, Bang et al. [99] studied milk yield and heat tolerance characteristics and revealed substantial regions on the BTA14 chromosome to be related to milk production characteristics. Macciotta et al. [97] studied milk production traits of milk yield, fat percentage, and protein percentage by applying principal component analysis (PCA). GWAS revealed that SNPs correlated with milk yield, fat, and protein percentage and with genes such as *HSF1* and *MCAT* related to heat tolerance [97].

Milk fatty acid (FA) composition exhibits huge variations between climatic conditions. Bohlouli et al. [100] aimed to identify candidate genes responsible for heat stress-altered milk fatty acid (FA) profiles using FA breeding values and temperature humidity index (THI) data. GWAS detected 30 SNPs connected to heat stress response (HSR) for different FA traits, emphasized the importance of FA in evaluating heat stress resilience, and implied genetic mechanisms for enhancing dairy cow tolerance to climate change. GWAS were also conducted in goats that identified candidate genes responsible for enhanced milk yield and content under heat stress [101]. Zidi et al. [101] reported significant SNPs responsible for tolerance to heat stress that occur within genes involved in milk composition, such as kappa casein (*CSN3*), acetyl–coenzyme A carboxylase alpha (*ACACA*), and malic enzyme 1 (*ME1*). Further, the GWAS for production traits under heat stress have also detected pathways that were influenced during stress conditions, such as pathways regulating metabolism, energy balance, and cellular stress responses. These pathways influence nutrient utilization and milk synthesis pathways, which will result in reduced production under adverse situations [6,100]. These findings indicate the utility of GWAS for genetic enhancement in thermo-tolerance-associated milk yield traits in livestock. Table 4 provides an overview of various candidate genes identified through GWAS governing production traits in ruminant livestock.

Further, it is also imperative to understand that heat stress also affects meat and wool production. As the feed intake is reduced during heat, the carcass quality, carcass quantity, and wool production are affected in meat- and wool-producing animals, like beef cattle and sheep. A GWAS involving beef cattle identified putative genes *LYN*, *PLAG1*, *WWOX*, and *PLAGL2*, which were found to influence growth and meat production traits in a tropical climate [121]. Similarly, studies have also been conducted in Nellore cattle, another well-adapted tropical breed [122]. In the study, the authors identified candidate genes and important metabolic pathways such as ABC transporter pathways, growth hormone synthesis, fatty acid metabolism, and insulin-signaling pathways that can be targeted to improve their productive potential. However, compared to milk production, there are limited studies that have focused on the genetic basis of the effect of heat stress on meat and wool production [122]. Therefore, more research is required in this field, as it can open new avenues to improve meat and wool production during heat stress conditions.

### 7.2. Reproduction Traits

Reproductive performance is one of the important economic traits in livestock. However, the rapid increase in temperature serves as a major obstacle for numerous reproductive processes, as it affects both the male and female reproductive systems, resulting in economic loss [123,124]. GWAS have helped to identify potential genes and uncover the complex genetic mechanisms behind the influence of heat stress on reproductive performance [102]. The GWAS has also helped underpin key pathways, such as the hypothalamic–pituitary–gonadal (HPG) axis, steroidogenesis, hormonal and cellular stress pathways, which are highly sensitive to thermal stress.

Heat stress decreases the fertility level of animals. An experiment on Pelibuey ewes under hot and humid environmental conditions detected seven thermo-tolerance SNP markers associated with reproductive and physiological characteristics [7]. The results showed that ewes with SNP markers rs421873172, rs417581105, and rs407804467 exhibited better heat stress than the control animals. These results suggest that these markers are for thermo-tolerance in Pelibuey ewes under semi-arid conditions [7]. Another experiment explored the potential of serum anti-Müllerian hormone (AMH) and associated SNPs as indicators of reproductive characteristics in Holstein cows under heat stress [102]. It identified four SNPs in the genes *AMH*, *LGR5*, *IGFBP1*, and *TLR4*, which were associated with AMH levels, reproductive characteristics, and physiological factors, and proposed that they are valuable biomarkers for enhancing fertility in cows under hot conditions [102]. Another study examined the effects of heat stress on heifer fertility characteristics and defined the critical time periods when heifers are most vulnerable to elevated environmental temperatures and humidity [103]. Substantial genotype-by-environment interactions (G × E) were found for three fertility traits being impacted by heat stress and highlighted the importance of considering G × E within breeding programs to enhance reproductive performance under stressful climatic conditions [103].

GWAS have also been performed to improve the fertility of males. A GWAS was performed on tropically adapted bulls to explore the molecular pathways of bull fertility and production. It detected several genes in BTA5 linked to spermatogenesis and male fertility traits [104]. Comparable research in the Nellore bulls characterized by climatic stress adaptation discovered significant genes on the X chromosome linked with male and female fertility and reproductive function [105]. It identified significant genes involved in oocyte maturation, thermogenesis, and sperm flagellum formation [105]. However, there have been very few studies aimed at enhancing male fertility under heat stress, warranting further research in this area. Table 4 provides an overview of various candidate genes identified through GWAS governing reproduction traits in ruminant livestock.

### 7.3. Immune Response Traits

Heat stress may impair immune function by interfering with both humoral and cell-mediated immune responses, resulting in increased susceptibility to various diseases. Such diseases can potentiate immune suppression caused by heat stress, which is modulated by the hypothalamic–pituitary–adrenal (HPA) and sympathetic–adrenal–medullary (SAM) axes [125,126]. Heat stress usually implicates pathways related to inflammatory regulation and protein stabilization and impacts the immune response in animals. In a GWAS study conducted by Carvalheiro et al. [8], various genes were found to be linked with environmental sensitivity in Nellore cattle. These genes were linked with biological processes such as inflammation, cell proliferation, immune response, and metabolism. Gene expression analysis showed that the potential genes were mainly expressed in the small intestine, adrenal gland, and pancreas, implicating their involvement in stress adaptation and immune regulation in cattle [8]. There are also similar association studies that have mapped potential candidate genes for immune responses to heat stress [127,128].

An increased risk of re-emerging and emerging pathogens and disease vectors is another effect of climate change [125]. The effect of prenatal heat stress on disease susceptibility in Holstein calves, with an emphasis on pneumonia (PNEU), diarrhoea (DIAR), and omphalitis (OMPH) [106], was assessed. The GWAS identified significant SNPs, involving 43 putative candidate genes with roles in biological processes, including viral life cycle regulation, immune response, and ubiquitination of proteins, suggesting their involvement in heat stress mechanisms and resistance to disease [106]. A similar study was conducted to investigate SNP × heat stress interactions for claw disorders in Holstein cows (dermatitis digitalis, interdigital hyperplasia, and sole ulcer) and identified several candidate genes linked to heat stress effects on claw health [107]. Hence, it is important to examine the interplay between heat stress and the immune response in the management of livestock subjected to heat stress. Yet, little has been explored regarding heat stress impacts on immune responses. Investigating further would be instrumental in curbing losses from disease outbreaks due to climate change and, more importantly, reducing further stress in animals due to diseases triggered by climate. Table 4 provides an overview of various candidate genes identified through GWAS governing immune response in ruminant livestock.

### 7.4. Adaptation Traits

GWAS are crucial for pinpointing adaptation traits in livestock, allowing breeders to boost resilience and productivity against environmental challenges. By scanning the genomes of large livestock populations, GWAS detect single-nucleotide polymorphisms (SNPs) that are linked to important adaptive traits, which are typically influenced by multiple genes [54].

Several GWAS have been conducted on livestock to identify potential genes and molecular pathways related to the response to heat stress. For example, Aboul-Naga et al. [9] conducted a study on different Egyptian sheep breeds, Saidi, Wahati, and Barki, with the aim of pinpointing genes associated with heat stress adaptation. Their research uncovered numerous SNPs associated with heat tolerance, particularly in genes associated with endoplasmic reticulum stress, thermoregulation, respiratory function, and melanin synthesis. These genes play a crucial role in maintaining normal biological processes, highlighting that heat tolerance is a significant function of these genes. The authors concluded Egyptian sheep to be superior to temperate breeds in unfavorable weather due to their genetic adaptation. Luna-Nevarez et al. [69] identified genetic markers associated with thermo-tolerance in pregnant Columbia × Rambouillet ewes in another study. They mapped SNPs in genes, like *FBXO11*, *PHC3*, *TSHR*, and *STAT1*, suggesting that these markers could be utilized in breeding programs to enhance the thermo-tolerance of sheep.

Using genome-wide association studies on 4662 cattle with varying degrees of indicine ancestry, Porto-Neto et al. [129] examined the genetic architecture of ten variables for tropical cattle production. The authors detected several important genes related to adaptation that could be introduced into temperate cattle breeds to improve their heat and disease resistance without reducing productivity. Additional studies in crossbred cattle (Bos taurus × Bos indicus), which are more heat-tolerant than taurine cattle, have also been conducted. For instance, Otto et al. [66] in their GWA analysis in crossbred cattle (Gir × Holstein) reported genes (*DGCR8*, *OSM*, *LIF*, *TXNRD2*) involved in thermal stress response in animals. In another study in Chinese Holstein cattle, the authors identified genetic markers associated with heat stress tolerance through a linear model-based GWAS. On three chromosomes, they identified seventeen SNPs, five of which were in the introns of genes *PDZRN4* and *PRKG1*, which had roles in blood vessel dilatation and protein degradation, respectively [108]. Furthermore, GWAS have also been conducted on buffaloes. In a study, the authors found that the Murrah buffaloes’ heat stress was linked to candidate genes (*FKBP5*, *FSHR*, *GRIN2A*, *SUGCT*, *NDRG*, and *TBC1D8*) [109]. In general, adaptability and thermo-tolerance potential in animals are regulated by pathways involving cellular stress responses, apoptosis, and thermoregulation mechanisms, enabling animals to cope with fluctuating environmental conditions. Thus, the above-discussed findings illustrate how GWAS unveil the genetic makeup of numerous economically relevant traits by identifying linked SNPs and pathways, which advances the molecular mechanism of heat stress response. To enhance breeding choices and increase livestock productivity and environmental stress resilience, these GWA studies assist in the identification of genetic markers. Table 4 provides an overview of various candidate genes identified through GWAS governing adaptive traits in ruminant livestock.

### 7.5. Low Methane Emission Traits

Methane (CH_4_) is a powerful greenhouse gas that contributes to 44% of global livestock emissions and 6% of anthropogenic greenhouse gas emissions. It is mostly produced by ruminant livestock through enteric fermentation and has a 21-fold higher potential for global warming than carbon dioxide [130,131,132]. Measuring and controlling methane emissions from cattle is important for the environment and for developing effective policymaking decisions. Genetic selection for low methane-emitting cows represents a sustainable strategy [110]. The genetic basis of methane emission features must be understood to develop sustainable breeding methods to reduce GHG emissions from livestock. However, the candidate gene approach has failed, highlighting the need for comprehensive genome-wide studies, such as GWAS, since CH_4_ emissions are complex features influenced by multiple genetic loci [133].

Manzanilla-Pech et al. [110] aimed to test the association of SNPs and genomic regions with eight methane emission traits in Danish Holstein cattle through GWAS. The study found that methane production and concentration are genetically closely related and share several significant genomic regions, particularly on chromosomes 13 and 26 [110]. Similarly, Calderon-Chagoya et al. [111] discovered a strong correlation between chromosome 13 and methane production. Calderon-Chagoya et al. [111] identified 46 SNPs that were previously associated with traits, such as feed intake, fatty acid content, and the composition of meat and milk, which were significantly linked to CH_4_ emissions. According to a different study, SNPs on BTA 14, more precisely in the *TRPS1* gene, were responsible for approximately 0.9% of the genetic variance for CH_4_ PPM/d and 1% for CH_4_ g/d in Polish Holstein–Friesian cows [112]. The identified SNPs explain only a small percentage of the genetic variance, suggesting that these traits are highly polygenic, and the investigators concluded that there may also be several other genes that influence CH_4_ methane production traits [112]. The VFA indicators are also known to enhance the prediction of PME traits, according to another GWA study that forecasted methane emission from volatile fatty acids in Iranian Holstein cattle [113]. The study discovered 33 significant SNPs and 69 connected genes linked to valeric acid and projected methane emissions (PMEs) [113].

**Figure 5:**
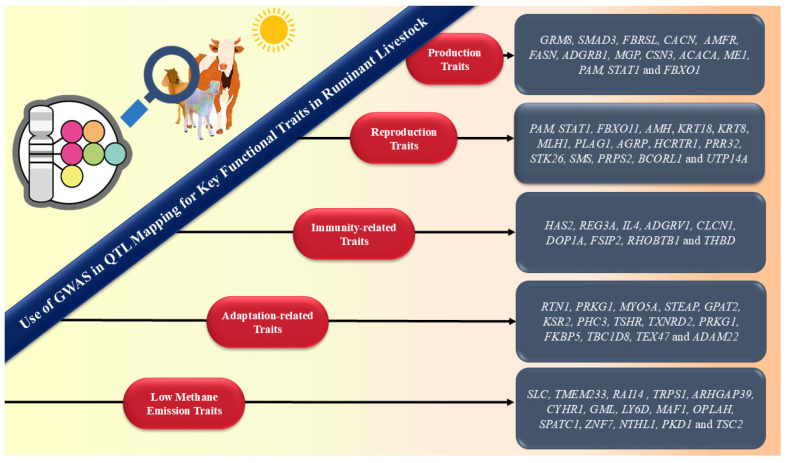
Description of applications of GWAS in QTL mapping for important economic traits.

Numerous genes associated with CH_4_ emission characteristics have also been identified through GWAS. The CH_4_ intensity and expected daily CH_4_ emissions in Walloon dairy cows were found to be associated with several putative candidate genes on chromosome 14 [114]. Similarly, Pszczola et al. [115] reported QTL regions and candidate genes, like *CYP51A1*, *NTHL1*, *PKD1*, *PPP1R16B*, and *TSC2*, which influence CH_4_ production and are linked to feed efficiency, milk traits, body size, and health in dairy cattle (Polish Holstein–Friesian cows). Remarkably, PKD1 was found to be an essential gene linked to the growth of the digestive system and potentially connected to the release of greenhouse gases, such as nitrogen and CH_4_ [115]. These GWA studies have, therefore, provided strategies for selecting genetically superior animals with lower methane production for more targeted breeding. However, since this field of study is still in its infancy, it requires immediate attention and ongoing development. Table 4 provides an overview of various candidate genes identified through GWAS governing immune response in ruminant livestock. Figure 5 describes the various applications of GWAS on QTL mapping.

### 7.6. GWAS-AI Model—An Integrated Modeling Approach for the Conservation of Indigenous Climate-Resilient Breeds

India, being one of the tropical countries, has a rich diversity of animal genetic resources, holding more than 70% of Earth’s biodiversity. It has a total of 161 well-recognized indigenous ruminant breeds: 53 breeds of cattle, 21 breeds of buffalo, 41 breeds of goat, and 46 breeds of sheep. These indigenous breeds are well adapted to the different agro-ecological zones and are known for their inherent climate-resilient potential and disease resistance. Therefore, in the face of climate change, it is important to conserve and utilize these genetic resources for improving livestock production while maintaining their genetic diversity. A sustainable way forward can be achieved by the integration of GWAS with advanced technologies, such as AI and precision livestock farming (PLF).

**Figure 6:**
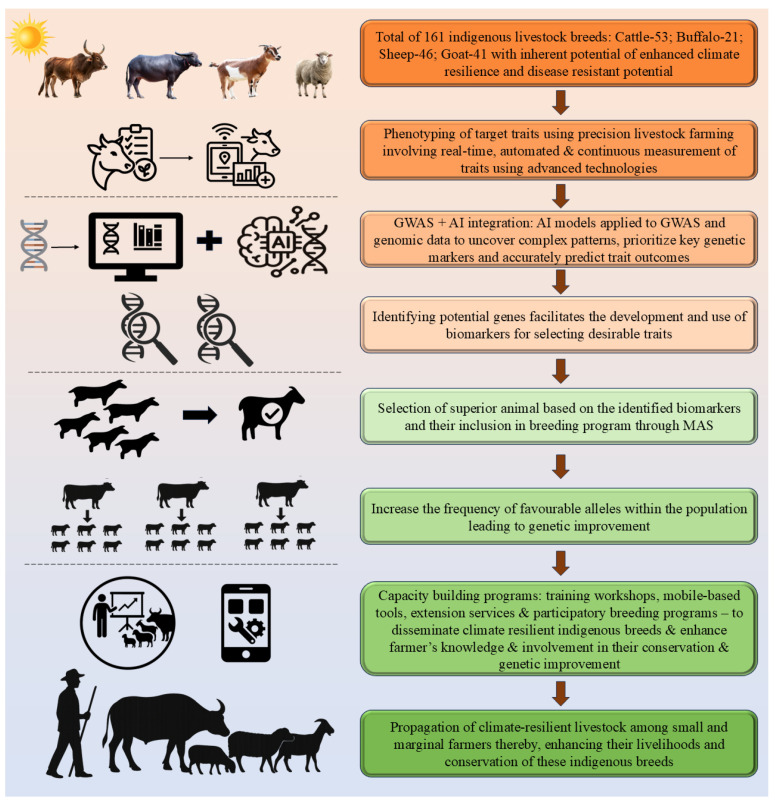
Conceptual GWAS-AI model for conserving the indigenous germplasms in livestock.

The effective utilization of GWAS depends on the availability of large, high-density datasets consisting of both phenotypic and genotypic data. The phenotypic data must be accurate and consistent to identify traits of economic and adaptive significance, which is possible with the help of PLF technologies. It is potentially one of the most powerful developments that is designed to monitor and control animal productivity, along with animal health and welfare parameters, in an automated manner. It aims to offer a real-time monitoring and management system for farmers. In parallel, large amounts of genotypic data are also necessary for the identification of genetic markers linked to targeted traits. The AI and ML algorithms, when applied to GWAS models and genomic datasets, can uncover complex molecular mechanisms, identify key genetic markers, and accurately predict trait outcomes. The genetic biomarkers can then be used to identify the animals with superior genetic merit and incorporate them into the breeding program through MAS. This enables the production of progenies with enhanced genetic potential. However, to ensure that the impact of these advancements reaches the farming community, it is essential that these genetically superior animals are disseminated to end-user farmers. This could be done through well-structured capacity building programs such as training workshops, mobile-based advisory tools, extension services, and participatory breeding programs. This will help in the adoption of indigenous breeds and enhance farmers’ knowledge, decision-making ability, and their long-term involvement in the conservation and genetic improvement of indigenous livestock resources. Thus, a synergistic multi-disciplinary approach (GWAS + AI + PLF) can facilitate data-driven breeding, real-time monitoring, and targeted interventions. Such an approach will not only result in enhanced productivity, climate resilience, and long-term sustainability of livestock production but also in the conservation and maintenance of the genetic diversity of these indigenous livestock breeds. Figure 6 describes the conceptual GWAS-AI model for conserving the indigenous germplasms in livestock.

## 8. Other Applications of GWAS in the Detection of Genomic Regions for Improving Climate Resilience in Livestock

Although heat stress significantly reduces animals’ productivity, their bodies adapt through various physiological processes to withstand extremely high temperatures and maintain survival [117,134]. The physiological mechanisms include rectal temperature, skin temperature, sweating rate, respiration rate, and pulse rate, which serve as valuable physiological indicators of heat stress. Furthermore, the underlying genetic variation in these physiological responses also influences the thermo-tolerant capacity of the animal [134]. Therefore, by selecting animals with favourable physiological indicators and better adaptive potential, it is possible to improve the climate resilience, productivity, and welfare of animals.

Body temperature, particularly rectal temperature, is a crucial physiological indicator for evaluating heat tolerance and overall health in livestock. GWAS focusing on rectal temperature have detected many SNPs and genomic regions that have a large effect on the rectal temperature. For example, Dikmen et al. [117] detected significant SNP variance on BTA24, with additional SNPs on BTA16, BTA5, BTA4, and BTA26 contributing to RT variation in Holstein cattle. In addition, they also detected candidate genes such as *SLC01C1*, *GOT1*, *KBTBD2*, *RFWD12*, *LSM5*, *SCARNA3*, *SNORA19*, and *U1* that were associated with RT [117]. In their similar follow-up study, they also found additional genes, *PGR* and *ASL*, to be associated with RT [118]. Similarly, ten significant SNPs on chromosomes BTA3, BTA4, BTA8, BTA13, BTA14, and BTA29 were found in recent research on Chinese Holstein cattle that evaluated rectal temperatures in the morning and afternoon [119]. Two genes, *FAM107B* and *PHRF1*, were identified by their gene expression analysis as important modulators of the heat stress response [119]. The authors adopted a weighted single-step GWAS (WssGWAS) in a follow-up study to include other physiological parameters, such as breathing rate, drooling score, and rectal temperature [54]. They identified 53 genomic areas linked to physiological heat stress indicators in Holstein cattle [54].

Another key sign of heat stress in animals is respiratory rate (RR). Animals that are under heat stress increase their respiration rate to activate their respiratory evaporative cooling systems [135]. Amamou et al. [136] observed that for every unit rise in THI, there was a corresponding increase of two breaths per minute. Sweating, which helps regulate internal body temperature, is another important evaporative cooling process during hot weather. Nunez-Soto et al. [116] investigated the relationship between SNP markers and heat stress resistance in Brown Swiss dairy cows using GWAS. Seven SNPs were significantly associated with respiratory frequency under heat stress, with key markers found on BTA 6, including SNPs within the *FAM13A* and *PI4K2B* genes, with the ARS-BFGL-NGS-102407 SNP on BTA 4 exhibiting the most associations. Numerous genes linked to metabolism, body condition, and protein synthesis have been identified, underscoring the genetic components that influence the Brown Swiss cattle in Yucatan, Mexico’s ability to withstand heat stress [116]. Further, SNPs were linked to both respiration and sweating rates as reported by Dikmen et al. [118]. *ACAT2* and *HSD17B7* explained variance in respiration rate, whereas *ARL6IP1* and *SERPINE2* accounted for most of the variation in sweating rate. It is noteworthy that *ARL6IP1* was linked to both sweating and respiration rates [118]. Hernandez et al. [120] performed a GWAS on Brangus cattle to find genetic loci linked to adaptation to heat stress. The study found quantitative trait loci (QTLs) on BTA7 and BTA12 that overlapped with the immune response and cellular proliferation-related genes *ADGRV1* and *CCDC168*. The authors suggested that these genes were probably involved in controlling heat tolerance and sweat gland activity [120]. Therefore, genetic selection that aims to increase animals’ tolerance to heat may benefit from these genetic markers. Table 4 describes the various candidate genes identified through GWAS governing productive, adaptive, and low methane emission in ruminant livestock.

## 9. Role of GWAS in Redefining Breeding Policies for Climate Resilience in Tropical Countries

Heat stress adversely affects livestock’s productivity, resulting in substantial economic losses [134]. Among the livestock species, dairy cows, due to the intensive breeding focused on enhancing milk production, are particularly vulnerable to heat stress, as it results in increased metabolic heat generation [137,138]. However, indigenous breeds, such as Zebu cattle in tropical regions, are well adapted to extreme heat stress, water scarcity, and reduced pasture availability while maintaining their productive and reproductive potential [1]. However, although they adapt to heat stress by lowering metabolic rate, heart rate, and increasing sweating capacity, their production levels are lower than those of exotic breeds [139].

**Figure 7:**
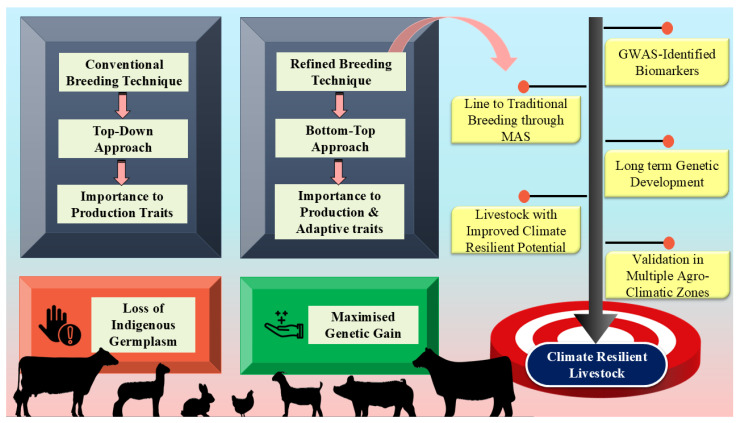
Proposed new breeding policy for climate-resilient livestock production.

Current crossbreeding policies in most tropical countries follow a top-down approach, primarily focusing on production traits instead of other important characteristics, such as climate and disease resistance [140]. Consequently, animal production increases, but they are unable to withstand harsh weather conditions, making them more susceptible to the effects of climate change. In addition, there has been a loss of indigenous cattle (Zebu cattle) germplasm that is known for their superior adaptability and acclimatization to harsh environmental conditions compared to crossbred and exotic breeds (Taurine cattle) [47,140]. Therefore, to improve livestock’s resilience to environmental problems, policymakers should think about moving away from the current top-down breeding technique and shift towards a bottom-up strategy that prioritizes not only production but also adaptability and disease resistance traits. This can be done using marker technology in breeding programs, in which the markers linked to genes controlling the desired phenotypes can be used to track and select animals in breeding programs. This will ensure the transmission of favorable alleles to the next generation. However, it is challenging to pinpoint the genes associated with adaptation traits because of their polygenic nature and the complex underlying mechanisms [141]. In this regard, GWAS enable the identification of candidate genes and genomic regions associated with indicators of the heat stress response, including variations that may have pleiotropic effects influencing multiple phenotypes related to heat stress [142]. GWAS enable the accurate identification of mutation locations, revealing novel single genes, by examining SNP/LD data at the individual or gene level. This provides valuable insights into the genetic mechanisms of heat tolerance, with the identified candidate genes serving as potential biomarkers for selection and breeding programs [4]. These biomarkers can be integrated into traditional breeding programs through marker-assisted selection. Marker-assisted selection (MAS) is a selection method in animal breeding that relies on genetic markers associated with desirable genetic traits. This selection method allows for the early selection of superior animal populations by choosing animals that have desirable alleles and improves the precision of selection compared to traditional selection methods. Such efforts enable breeders to design breeding programs that maximize genetic gains across various traits, including productivity, disease resistance, and heat tolerance capacity in animals [128]. Additionally, the practical application of the findings of GWAS can be achieved using identified SNPs or genes for heat tolerance and adaptability in the selection indices. For example, SNP markers for heat tolerance and adaptability traits, such as rectal temperature or milk production under heat stress, can be used for genomic selection of heat-tolerant breeds. Similarly, haplotypes or QTL for heat tolerance and adaptability identified through GWAS for adaptive traits can be used for marker-assisted or genomic selection of heat-tolerant breeds with high production potential. Thus, GWAS can play a pivotal role in refining breeding policies to select animals with improved climate resilience. However, before breeding policies are refined and incorporated into genetic selection programs, the GWAS-identified markers need to be validated in independent agro-climatic zone-specific livestock populations [1]. Figure 7 describes a new breeding policy involving GWAS for climate-resilient livestock production.

Additionally, utilizing GWAS in breeding programs also has the important advantage of promoting long-term genetic development as selection based on genetic biomarker results in cumulative and durable genetic gains [47,143]. The incorporation of genetic markers into conventional breeding systems improves the accuracy and precision of heat tolerance selection without jeopardizing the animal’s productive potential [142]. As a result, the GWAS is a useful tool that can help refine breeding policies and implement effective strategies for developing heat-tolerant livestock that can produce as well as adapt optimally, presenting long-term solutions to heat stress. Such strategies are necessary to mitigate the consequences of climate change and enhance livestock production to satisfy the demands of the world’s expanding population.

## 10. Challenges and Limitations of GWAS in Livestock

**Figure 8:**
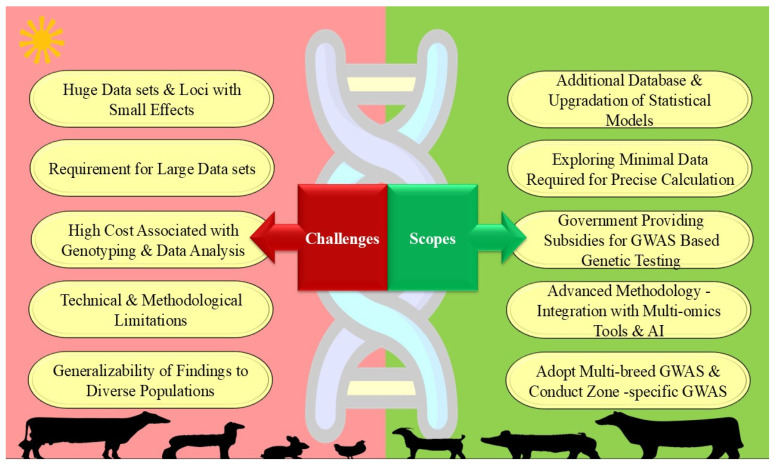
Various scopes and challenges associated with GWAS for climate-resilient livestock production.

The literature and numerous research studies offer examples of GWAS explaining the genetic basis and underlying molecular mechanism of various economic traits [144]. However, the effectiveness of GWAS is hindered by constraints such as large datasets, missing genotypes, genetic variability, low linkage disequilibrium (LD), small effect sizes, low allele frequencies, and the complex genetic architecture of traits [144,145]. Figure 8 describes the various scopes and challenges associated with GWAS for climate-resilient livestock production.

The main drawback of GWAS is the requirement for huge datasets from a homogeneous population [11,146], especially when identifying loci with small effects. This is particularly challenging in tropical and sub-tropical regions where farming is not commercialized and animals are often from multiple farms. In many GWAS, the identified candidate genes and genomic regions related to heat stress generally exhibit a small effect size [128], highlighting that adaptation is a highly polygenic and complex trait. This makes it difficult to detect significant associations and necessitates further investigation to assess the pleiotropy between heat stress response indicators and other traits related to production and reproduction to evaluate the significance of the variants [128]. This highlights the importance of a collaborative approach with different fields, i.e., statisticians and bioinformaticians, to expand the research of such complex traits in livestock, which will require additional database and statistical model upgrades to improve precision and accuracy [147]. Furthermore, the ability of GWAS to accurately identify the genetic determinants of thermo-tolerance traits is limited by factors such as epistasis, epigenetics, microbiota, and environmental impacts that contribute to missing heritability [148]. These issues can be resolved by including non-additive effects in GWAS models that can help address the problem and improve the identification of climate resilience loci [149].

Moreover, GWAS in livestock encounter issues such as the impact of management practices, environmental variables, and breed variations on the outcomes [146]. Findings from one breed or location may not apply to others, limiting the generalizability of the findings to diverse populations [4]. Further, the use of crossbred animals can complicate the interpretations even more due to differing genomic regions and allelic frequencies. Thus, caution is necessary when applying results from crossbred animals to purebred populations, as genetic backgrounds can significantly influence heat stress response associations [128,146].

The inability to record phenotypic data is another major obstacle in employing GWAS in developing nations, as most of the livestock farmers in tropical regions are small-scale operators adhering to traditional and extensive production systems. These farmers often face challenges in systematically documenting and recording the phenotypic data [150]. As a result, the data collection can be inconsistent, leading to biases in the results that make it difficult to apply advanced genetic tools like GWAS effectively. In these circumstances, where gathering data is a significant limitation, it is essential to explore the minimal amount of information needed to precisely forecast an animal’s genetic merits. This allows data collection systems to be simplified while continuing to provide accurate results [151].

Additionally, several technical and analytical problems plague GWAS, such as population stratification, limited detection power, missing loci, repeated test corrections, and trouble detecting uncommon variants and CNVs [144]. Methodological problems such as poor study design, data structure, genotyping errors, and phenotyping uncertainty increase the risk of Type I and Type II errors, limiting the accuracy of genetic variance estimation for complex adaptation traits [149]. Furthermore, in developing nations, access to genomic breeding methods may be limited due to the high costs associated with genotyping, data analysis, and infrastructure [152,153]. Therefore, even though GWAS make it possible for sophisticated methods like gene editing and genetic selection, cost, regulatory, and social constraints limit their use [153]. Lastly, there are also ethical questions about animal care and selective breeding in the application of genetic markers in animal breeding [153].

## 11. Conclusions

**Figure 9:**
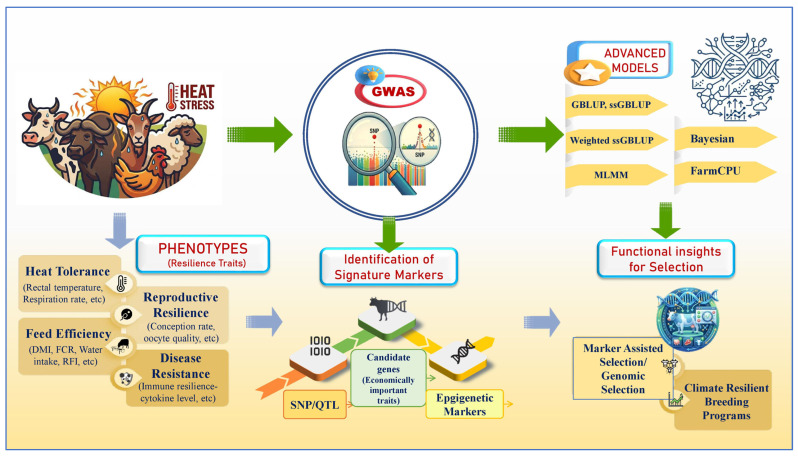
Application of GWAS in enhancing climate resilience in livestock.

The challenging climatic conditions and the projected changes urgently demand effective mitigation strategies and adaptation practices. One well-established approach is the use of genetic tools to identify climate-resilient livestock breeds. As livestock resilience varies among species, breeds, and agro-climatic regions, it prompts a significant need to identify climate-resilient breeds suited to each agro-climatic zone. The GWAS has emerged as a powerful molecular tool with wide practical applications in identifying genetic variants linked to various complex traits, following the availability of genome sequences and the identification of SNPs. The scientific evidence demonstrates the potential of GWAS in QTL mapping for production, reproduction, immune response, adaptation, and low methane emission traits, thereby unveiling their genetic makeup. This has facilitated selecting animals through marker-assisted and genomic selection by identifying those with desirable physiological traits and better adaptability. These insights support the development of climate-resilient breeding programs by improving selection accuracy and accelerating genetic gain under environmental stress conditions. Additionally, integrating GWAS with multi-omics approaches (e.g., transcriptomics, epigenomics) and AI, ML, and DL algorithms presents scalable applications and is a promising approach for developing more effective climate-resilient breeding strategies. The potential of GWAS to pinpoint genes associated with adaptation traits in addition to production traits is a vivid testament to its competitive edge over other technologies. While GWAS for adaptive traits have limitations, including low heritability, need for big datasets, genetically homogenous population, low linkage disequilibrium (LD), small effect sizes, low allele frequencies, complex trait architecture, and other technical, analytical, social, and ethical constraints, their potential value in redesigning breeding programs is considerable. The GWAS-based identification of biomarkers could provide a foundation for future breeding efforts. By promoting genetic selection based on these biomarkers, the GWAS tool supports long-term genetic improvement and offers a sustainable solution for breeding climate-resilient livestock. Figure 9 provides an overview of the application of GWAS in enhancing climate resilience in livestock.

## 12. Future Perspectives

**Figure 10:**
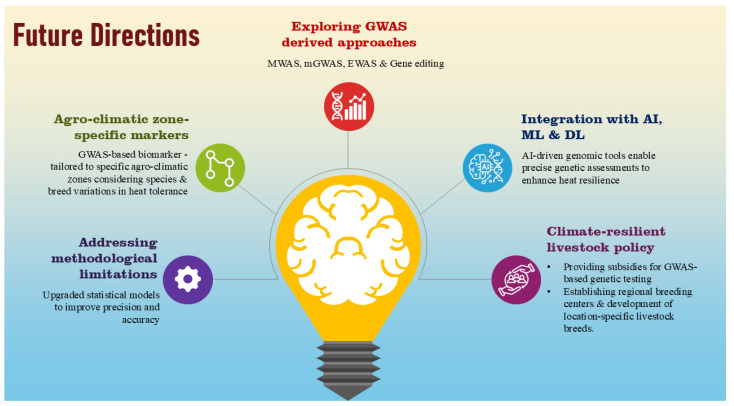
Applications of GWAS for climate-resilient livestock production in the future. MWAS, microbiome-wide association studies; mGWAS, microbiome genome-wide association studies; EWAS, epigenome-wide association studies; AI, Artificial Intelligence; ML, machine learning; DL, deep learning.

Given the plausible role of GWAS in identifying climate-resilient livestock breeds, their reported challenges are often outweighed. Primarily, future studies addressing the methodological limitations of GWAS are essential, including developing alternatives for challenges related to study population, statistical model, and other technical, analytical, economic, and ethical issues. Research pertaining to optimizing livestock breeding programs and enhancing resilience to challenging climates using the GWAS tool is still in its infancy. Future studies involving GWAS-based biomarker identification should be tailored to specific agro-climatic zones across various livestock species and breeds, considering variations in heat-tolerance. A critical gap in the literature is the lack of functional validation of the potential biomarkers for inducing climate resilience. Hence, future research efforts are warranted in addressing this limitation to fully harness their potential in developing robust, climate-resilient breeds that could adapt to multiple agro-ecological zones, produce optimally, and emit less methane per unit of feed consumed. Figure 10 describes the future applications of GWAS for climate-resilient livestock production.

Another exciting path for future research involves deeper exploration of GWAS-derived approaches, including microbiome-wide association studies (MWAS), microbiome genome-wide association studies (mGWAS), epigenome-wide association studies (EWAS), and gene editing (CRISPR/Cas9) methods to reveal novel mechanisms underlying phenotypic variation. In addition, following the technological bloom, AI-enabled GWAS analysis has become the mainstream research priority. To enhance the effectiveness of developing climate-resilient livestock herds, AI-enabled technologies should be coupled with GWAS, and this demands more iterative experimentation in the future. The potential of GWAS-derived biomarkers for ensuring climate resilience can be fully harnessed only if researchers, decision-makers, funders, and governments join forces. Governments should prioritize the conservation of local genetic resources and support climate-resilient livestock breeding by providing subsidies for GWAS-based genetic testing, establishing regional breeding centres, and promoting the development of location-specific livestock breeds. These recommendations can be crucial guidelines for future studies and represent a sustainable solution to ensure climate-resilient livestock herds for future generations.

## Figures and Tables

**Table 1:** Summary of genetic models for establishing climate resilience in livestock.

Genetic Model	Key Characteristics	Advantages	Disadvantages/Limitations	References
GBLUP (Genomic Best Linear Unbiased Prediction)	Uses a genomic relationship matrix (GRM) derived from SNP data to estimate genomic breeding values (GEBVs)	Higher accuracy than pedigree-based models; suitable for genomic selection; effective for heat tolerance traits	Assumes equal SNP effects; may not capture complex genetic architecture fully	[48,49,50]
ssGBLUP (Single-step GBLUP)	Integrates pedigree, phenotypic, and genomic data simultaneously	Improved accuracy and reduced bias; efficient use of all available data	Computationally demanding; requires high-quality integrated datasets	[51,52,53]
Weighted ssGBLUP/WssGWAS	Assign weights to SNPs based on their effects; improves GRM	Better detection of QTL; increased prediction accuracy; accounts for marker importance	Slight improvement over ssGBLUP; iterative weighting increases complexity	[54,55]
GLMM (Generalized Linear Mixed Model)	Extends GLM by including fixed and random effects; handles non-normal data	Suitable for longitudinal and clustered data; accounts for environmental variation	Model complexity; computational intensity; requires careful model specification	[56,57]
Random Regression Models (RRM)	Models genetic and environmental effects over time; use reaction norms	Captures dynamic responses to heat stress; evaluates phenotypic plasticity	Requires large datasets; complex interpretation	[58,59,60]
GLM (General Linear Model)	Simple linear model without random effects	Easy to implement; low computational demand	Cannot control population structure; prone to false positives	[61]
MLM (Mixed Linear Model)	Includes both fixed and random effects; controls population structure	Reduces false positives; widely used in GWAS	Computationally intensive; may miss small-effect loci	[63,64,65]
Single-SNP GWAS Model	Tests one SNP at a time for association	Simple and interpretable; widely used	Ignores polygenic nature; high false discovery risk	[66]
Single-locus Mixed Model	Similar to single-SNP but includes random effects	Controls confounding factors better than single-SNP	Limited power for complex traits	[67]
Bayesian Models (e.g., Bayes B, Bayes C)	Multi-locus models estimating all marker effects simultaneously with prior distributions	Captures polygenic traits; flexible; effective for variable selection	Computationally intensive; requires prior assumptions	[68,69]
PCA-based GWAS	Uses principal components to analyze multiple correlated traits	Increases statistical power; detects pleiotropic QTLs	Interpretation can be complex; may lose trait-specific detail	[62]
FarmCPU (Fixed and Random Model Circulating Probability Unification)	Iterative model combining GLM and MLM; separates testing markers and covariates	Reduces false positives and negatives; high statistical power	Requires careful parameter tuning; computationally intensive	[70,71]
Multi-locus Models (SUPER, MLMM, Blink)	Consider multiple loci simultaneously	Better detection of true associations; improved control of false positives	Higher computational requirements; model complexity	[61]

**Table 2:** Various GWAS models used for identifying biomarkers governing various functions using different whole-genome sequencing.

Genetic Model	Species	Breed	Trait	Chip	Reference
Random regression model	*Bovine*	Holstein and Jersey	Milk production	Illumina Bovine High-Density genotype	[49]
Random regression model	*Bovine*	Holstein cows	Milk production	-	[50]
Single-step genomic best linear unbiased prediction; linear regression model	*Bovine*	Thai Holstein cows	Milk yield	Illumina BovineSNP50	[52]
Weighted single-step genome-wide association study	*Bovine*	Holstein cows	Rectal temperature, respiration rate, and drooling score	Illumina 150 K Bovine Bead chip	[54]
Generalized linear mixed model	*Bovine*	Holstein cows	Reproduction performance	-	[57]
Random regression model	*Bovine*	Multi-breeds	Milk production	-	[60]
Mixed linear model	*Ovine*	Chios dairy sheep	Milk yield	OvineSNP50 Genotyping	[65]
Single-SNP GWAS	*Bovine*	Gir × Holstein F2 population	Rectal temperature	Illumina BovineSNP50v1	[66]
Single locus mixed model GWAS	*Ovine*	Greek sheep	Adaptation	Illumina Ovine 50K SNP	[67]
Single-marker analysis and Bayesian multi-marker models	*Ovine*	Columbia–Rambouillet crossbred ewes	Adaptation	Illumina OvineSNP50 BeadChip	[69]
FarmCPU	*Bovine*	Holstein cows	Fertility	-	[71]

**Table 3:** Comparison of genomic models and AI/ML approaches for predicting phenotypes and climate adaptability in livestock.

Approach	Key Characteristics	Applications in Climate Adaptability	Advantages	Limitations/Disadvantages	References
Genomic models (reaction norms, GWAS, G × E models, omics integration)	Use SNPs, genomic markers, and reaction norm models to evaluate genotype–environment interactions (G × E); rely on statistical genetics frameworks	Identification of biomarkers for thermo-tolerance; estimation of phenotypic plasticity; detection of adaptive genes; selection of climate-resilient breeds	Biologically interpretable; directly links genotype to phenotype; robust for detecting QTLs and G × E interactions; supports marker-assisted selection (MAS)	Requires large, high-quality genomic datasets; limited ability to capture non-linear relationships; moderate predictive power for complex traits	[73,74,75,76,77,78,79,80]
Reaction norm models (within genomic approaches)	Model phenotypic response across environmental gradients	Evaluation of heat tolerance and adaptive responses under varying THI; identification of resilient genotypes	Captures environmental sensitivity; improves accuracy of G × E estimation	Data-intensive; complex modeling and interpretation	[75,79]
Omics-based genomic approaches (RNA-seq, microarrays, GWAS integration)	Integrate transcriptomics and genomics to study gene expression under stress	Identification of genes regulating adaptive responses; understanding the molecular basis of plasticity	High-resolution biological insight; identifies regulatory pathways	Expensive; complex data integration; requires bioinformatics expertise	[78,80]
AI/machine learning models	Use algorithms to analyze large, high-dimensional datasets; detect patterns beyond linear assumptions	Prediction of adaptive traits; genomic prediction of heat tolerance; integration of environmental and genomic data; decision support in breeding	Handles big data efficiently; captures non-linear relationships; higher predictive accuracy; adaptable to multi-source data	Risk of overfitting; limited biological interpretability (“black box”); requires large datasets and computational power	[12,13,14,86,88]
Deep learning (DL)	Advanced ML using neural networks for feature extraction and pattern recognition	Analysis of NGS data; prediction of complex adaptive traits; integration of multi-omics and environmental data	Superior performance in complex datasets; automatic feature extraction	Poor generalization across datasets; high computational cost; interpretability challenges	[88,92]
AI-integrated genomic prediction models	Combine ML with genomic selection (genomic + phenotypic + environmental data)	Improved prediction of breeding values for thermo-tolerance and productivity under climate stress	Enhanced prediction accuracy; faster decision-making; supports precision breeding	Data integration challenges; requires validation across populations	[86,89]
Machine learning for selection signatures	Treats selection detection as a classification problem	Identification of adaptive genomic regions under climate stress	More robust than traditional statistical assumptions; handles complex patterns	Requires labelled datasets; methodological complexity	[90,91]

**Table 4:** Various candidate genes identified through GWAS governing productive, adaptive, and low methane emission in ruminant livestock.

Traits	Species	Breed	Parameters	Candidate Genes/Genomic Regions	Functions	Reference
Productions traits	*Bovine*	Holstein cows	Total milk yield, rectal temperature, and respiration rate	*TLR4*, *GRM8*, and *SMAD3*	Immune response, glutamate release, regulation of lipid and adipose tissue metabolism	[6]
	*Bovine*	Exotic Holstein and Jersey; crossbreds; native—Hallikar and Khillar	Test day milk yield	*FBRSL* and *CACN*	Immune response, cell signaling, ubiquitination	[98]
	*Bovine*	Vietnamese dairy cattle	Milk yield, energy corrected milk yield, fat%	BTA14		[99]
	*Bovine*	Italian Holstein cows	Milk yield, fat%, protein%	*HSF1*, *MCAT*	Regulation of stress proteins, fatty acid metabolism	[97]
	*Bovine*	Holstein cows	PUFA, SFA	*AMFR*, *FASN*, *ADGRB1*, and *MGP*	Fatty acid synthesis and disease resistance	[100]
	*Caprine*	Florida goats	Milk composition	*CSN3*, *ACACA*, *ME1*	Regulation of cell death in the mammary gland and thermo-tolerance function of HSP27	[101]
Reproduction	*Ovine*	Pelibeuy ewes	Conception rate, Lambing rate	*PAM*, *STAT1*, and *FBXO11*	Modulates the effects of HSPs	[7]
	*Bovine*	Holstein cows	AMH level	*AMH*, *LGR5*, *IGFBP1*, and *TLR4*	Regulation of follicular growth, cell proliferation and survival, immune response	[102]
	*Bovine*	Holstein heifers	Age at first calving, service period, conception rate	*SP1*, *KRT18*, *KRT8*, *MLH1*, *EOMES*, *PLAG1*, *AMHR2*, *PC*, *AGRP*, *GUCY1B1*, and *HCRTR1*	Oocyte development, maturation, differentiation, embryo development, oogenesis, stress response regulation	[103]
	*Bovine*	-	Scrotal circumference, sheath score	*DYRK2*, *GRIP1*, *CAND1*	Spermatogenesis	[104]
	*Bovine*	Nellore	18 male and 5 female fertility and reproductive traits	*PRR32*, *TMSB4X*, *STK26*, *TLR7*, *SMS*, *PRPS2*, *SMARCA1*, *BCORL1*, *UTP14A*	Oocyte maturation, thermogenesis, sperm flagellum development, immune response	[105]
Immune response	*Bovine*	Nellore		*HAS2*, *REG3A*, and *IL4*	Inflammation, cell proliferation, immune response, and metabolism	[8]
	*Bovine*	Holstein calves		43 candidate genes	Viral life cycle regulation, immune response, and protein ubiquitination	[106]
	*Bovine*	Holstein cows		*ADGRV1*, *CLCN1*, *DOP1A*, *FSIP2*, *RHOBTB1* and *THBD*	Blood clotting, growth hormone and prolactin secretion, electrical excitability of skeletal muscles	[107]
Adaptation	*Ovine*	Saidi, Wahati, and Barki	Animal Heat Tolerance Index	*RTN1*, *PRKG1*, *GSTCD*, *MYO5A*, *STEAP3*, *GPAT2*, *KSR2*	Endoplasmic reticulum stress, thermoregulation, respiratory function, and melanin production	[9]
	*Ovine*	Columbia × Rambouillet ewes	Thermotolerance indicator	*FBXO11*, *PHC3*, *TSHR*, and *STAT1*	Regulation of HSPs	[69]
	*Bovine*	Gir × Holstein	Rectal temperature	*DGCR8*, *OSM*, *LIF*, *TXNRD2*	Regulation of HSP, RNA degradation, redox regulation	[66]
	*Bovine*	Chinese Holstein	Rectal temperature, drooling score, and respiratory score	*PDZRN4* and *PRKG1*	Protein degradation and blood vessel dilation	[108]
	*Buffalo*	Murrah	Test day milk yield	*FKBP5*, *FSHR*, *GRIN2A*, *SUGCT*, *NDRG*, *TBC1D8*	Regulation of various metabolic and biological pathways	[109]
	*Ovine*	Greek sheep breeds		*TEX47*, *SRI*, *STEAP4*, *ZNF804B*, and *ADAM22*	Photoreceptor activity, maintenance of cellular homeostasis	[67]
Low methane emission	*Bovine*	Danish Holstein cows	Methane concentration (MeC), methane production (MeP)	Genomic regions, particularly on chromosomes 13 and 26	-	[110]
	*Bovine*		Methane production	*SLC23A2*, *SLC9A9*, *TMEM233*, *RAI14*	Milk fatty acid composition	[111]
	*Bovine*	Polish Holstein–Friesian	CH_4_ ppm/d, CH_4_ g/d	BTA 14—*TRPS1* gene	Regulates milk fat yield	[112]
	*Bovine*	Italian Holstein	Predicted methane emissions (PME) and valeric acid traits	40 and 17 significant SNPs for PME and valeric acid, respectively	Organelle organization and olfactory receptor activity	[113]
	*Bovine*	Walloon dairy cows	CH_4_emissions and methane emission intensity	*ARHGAP39*, *CYHR1*, *GML*, *LY6D*, *MAF1*, *OPLAH*, *PPP1R16A*, *SPATC1* and *ZNF7*	Methane emissions	[114]
	*Bovine*	Polish Holstein–Friesian	CH_4_ production	*CYP51A1*, *NTHL1*, *PKD1*, *PPP1R16B*, and *TSC2*	Digestive development and potential involvement in GHG emissions	[115]
Physiological response traits	*Bovine*	Brown Swiss	Respiratory frequency	*FAM13A* and *PI4K2B*	Protein production, body condition, and metabolism	[116]
	*Bovine*	Holstein	Rectal temperature	*SLC01C1*, *GOT1*, *KBTBD2*, *RFWD12*, *LSM5*, *SCARNA3*, *SNORA19*, and *U1*	Protection from cellular stress, RNA metabolism, protein ubiquitination	[117]
	*Bovine*	Holstein	Rectal temperature	PGR, *ASL*, *ARL6IP1*	Progesterone regulation, inhibits apoptosis	[118]
	*Bovine*	Chinese Holstein	Rectal temperature	*FAM107B* and *PHRF1*	Repair cell damage	[119]
	*Bovine*	Chinese Holstein	Rectal temperature, respiration rate, and drooling score	*PMAIP1*, *SBK1*, *TMEM33*, *GATB*, *CHORDC1*, *RTN4IP1*, and *BTBD7*	Autophagy regulation, heat shock, and cellular adaptive functions, maintenance of homeostasis	[54]
	*Bovine*	Holstein	Respiration rate	*ACAT2* and *ARL6IP1*	Inhibits apoptosis	[118]
	*Bovine*	Holstein	Sweating rate	*ARL6IP1* and *SERPINE2*	Inhibits apoptosis, inhibits thrombin, and plasminogen activator	[118]
	*Bovine*	Brangus cattle	Sweat gland traits	*ADGRV1* and *CCDC168*	Immune response and cellular proliferation	[120]

## Data Availability

No new data were created or analyzed in this study. Data sharing is not applicable to this article.
